# Post-Moore two-dimensional integrated electronics for angstrom-nodes

**DOI:** 10.1093/nsr/nwag166

**Published:** 2026-03-18

**Authors:** Zizhuo Shen, Yihao Zheng, Fansheng Peng, Yongfeng Wang, Lian-Mao Peng, Chenguang Qiu

**Affiliations:** Key Lab for the Physics and Chemistry of Nanodevices and Center for Carbon-based Electronics, School of Electronics, Peking University, Beijing 100871, China; Key Lab for the Physics and Chemistry of Nanodevices and Center for Carbon-based Electronics, School of Electronics, Peking University, Beijing 100871, China; Academy for Advanced Interdisciplinary Studies, Peking University, Beijing 100871, China; Key Lab for the Physics and Chemistry of Nanodevices and Center for Carbon-based Electronics, School of Electronics, Peking University, Beijing 100871, China; Key Lab for the Physics and Chemistry of Nanodevices and Center for Carbon-based Electronics, School of Electronics, Peking University, Beijing 100871, China; Key Lab for the Physics and Chemistry of Nanodevices and Center for Carbon-based Electronics, School of Electronics, Peking University, Beijing 100871, China; Key Lab for the Physics and Chemistry of Nanodevices and Center for Carbon-based Electronics, School of Electronics, Peking University, Beijing 100871, China

**Keywords:** post-Moore, two-dimensional materials, angstrom-nodes, next-generation transistors, scaling limits

## Abstract

As integrated circuits approach the angstrom-nanometer nodes in recent years, the continued downscaling of silicon-based electronics faces fundamental physical limits and escalating manufacturing costs. Two-dimensional (2D) materials—characterized by their atomic thickness, exceptional electrostatic gate control, and inherent suitability for heterogeneous integration—have arisen as promising alternatives to extend device scaling beyond the limits of silicon technology. This review provides a systematic overview of advances in integrated electronics based on 2D materials. The discussion begins with recent developments in wafer-scale synthesis and transfer techniques for 2D semiconductors. Subsequently, progress in high-performance 2D transistors is discussed, focusing on gate dielectric integration, contact optimization, and scaling strategies for high-density and advanced-node configurations. Circuit- and system-level implementations are also surveyed, including logic, memory, and three-dimensional (3D) monolithic integration. Further, key challenges and prospective directions are highlighted for realizing scalable, manufacturable, and low-power 2D electronic systems at the angstrom-nodes.

## Teaser

This comprehensive review systematically outlines key advances in two-dimensional technology that enable post-silicon angstrom-node integrated electronics.

## INTRODUCTION

Since Gordon Moore proposed in 1965 that the number of transistors on an integrated circuit would double approximately every two years—known as Moore’s Law [1]—silicon-based microelectronic devices have driven decades of rapid growth in the semiconductor industry. However, as transistor dimensions enter the nanometer regime, channel lengths continue to shrink and the device dimensions keep compressing, silicon-based MOSFETs increasingly approach their physical limits. The performance, power consumption and integration of silicon-based transistors are gradually approaching their limits [2].

**Figure 1.**
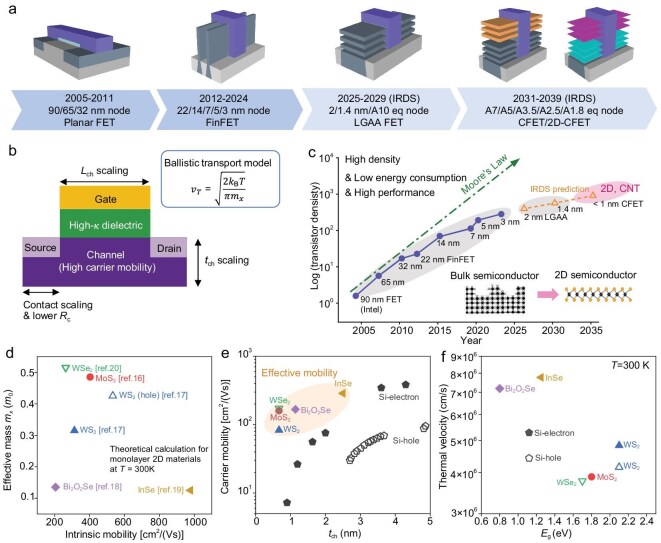
Evolution of silicon integrated circuit nodes and performance of 2D semiconductor materials. (a) The development trend of transistor structures. Since the planar FET of 32 nm technology node, transistor structures have evolved with technology nodes: FinFET structures were introduced at the 22 nm node; according to IRDS predictions, LGAA structures will be adopted at the 2 nm node, and CFET (Complementary FET) architectures will be implemented at angstrom nodes. (b) Key parameters in the process of transistor scaling down. (c) The scaling trend of transistors, including Moore’s Law and IRDS predictions. CNT: carbon nanotube. (d) Theoretical calculated intrinsic material mobility and effective mass of several 2D semiconductors at room temperature. Solid symbols represent electron mobility, while open symbols represent hole mobility. (e) Carrier mobility as a function of channel thickness for different semiconductor channel materials at room temperature. For silicon, electron and hole mobilities decrease sharply below 4 nm [21,22]. In contrast, 2D materials such as MoS_2_ [10], WS_2_ [23], WSe_2_ [24], InSe [25], and Bi_2_O_2_Se [26] maintain high mobility even at atomic layer thickness in the experiment. (f) Bandgap and carrier thermal velocity of silicon and 2D semiconductors at room temperature. Solid symbols denote electron thermal velocities, while open symbols denote hole thermal velocities.

To mitigate the degradation of gate control caused by short-channel effects [2], novel device architectures, including FinFETs [3,4], lateral gate-all-around (LGAA) FETs [5, 6], and complementary field-effect transistors (CFETs) [7] have been proposed, as illustrated in Fig. 1a. These structures expand the effective gate control area, significantly enhancing the electrostatic control of silicon transistors. Figure 1b illustrates that during transistor scaling, multiple key parameters must be systematically optimized: channel length (*L*_ch_) and thickness (*t*_ch_) should be co-designed with the decrease in contact length to maintain electrostatic integrity. Meanwhile, adopting high-mobility channel materials enhances carrier transport efficiency, while integrating high-*κ* dielectric layers improves gate control capability, thereby optimizing subthreshold swing (SS) characteristics. Additionally, reducing contact resistance is critical for preserving device performance. When the channel length is scaled below 10 nm, the carrier transport mechanism transitions from diffusion-dominated to ballistic transport [8, 9], where scattering effects are significantly suppressed, pushing device performance closer to theoretical limits. Concurrently, emerging two-dimensional (2D) materials offer a new avenue to surpass the physical limits of silicon, exhibiting potential to complement or replace conventional silicon-based devices [10, 11]. According to International Roadmap for Devices and Systems (IRDS)’s prediction [12], by 2035, advanced transistor nodes will reach angstrom dimensions, with CFET logic architectures and novel semiconductor materials such as 2D materials and IGZO entering large-scale fabrication, as illustrated in Fig. 1c.

In transistors scaled proportionally in the lateral dimension, the channel thickness must also be reduced to maintain sufficient gate electrostatic control for effective device switching. However, conventional three-dimensional semiconductors (e.g., Si and Ge) exhibit significant mobility degradation when thinned to the nanometer scale due to enhanced surface scattering [13]. In contrast, 2D layered semiconductors with atomically flat intrinsic surfaces provide a unique solution. These materials naturally possess atomic-scale thickness and low surface roughness, enabling high carrier mobility while satisfying electrostatic control requirements under scaling [14, 15]. Figure 1d shows the calculated intrinsic material mobility and effective mass of several commonly studied 2D semiconductors from first-principles calculations [16–20]. These intrinsic properties, primarily governed by lattice phonon scattering, underscore the strong potential of 2D materials for high-performance nanoscale electronic devices. Figure 1e depicts the variation of electron/hole effective mobility of 2D materials with thickness scaling, where silicon [21, 22] exhibits a sharp mobility decline below 4 nm due to surface carrier scattering. The effective mobility refers to the experimentally extracted value in practical devices and is modulated by extrinsic factors such as interface scattering, defect scattering, and contact resistance. Experimental results demonstrate that when the equivalent oxide thickness (EOT) is <3 nm, monolayer or ultrathin (≤1 nm) 2D materials—including MoS_2_ [10], WS_2_ [23], WSe_2_ [24], InSe [25], and Bi_2_O_2_Se [26]—exhibit significantly higher mobility compared to silicon-on-insulator (SOI) or bulk silicon of equivalent thickness.

When the channel length is reduced to below 10 nm, the transport mode of the transistor is determined by the ballistic transport model [8, 9], rather than the traditional drift-diffusion mechanism. Under high *V*_DS_ bias voltage, the ballistic current is determined by the carrier injection velocity emitted from the source over the top of the potential barrier, $\sqrt {\frac{{2{k}_{\mathrm{B}}T}}{{\pi {m}_x}}} $, where *k*_B_ is the Boltzmann constant, *T* is the lattice temperature, and *m_x_* is the effective mass along the transport direction. Figure 1f illustrates the relationship between bandgap and thermal velocity for Si and several 2D materials [10, 18, 27–30]. At room temperature, InSe and Bi_2_O_2_Se exhibit smaller effective masses, leading to higher carrier thermal velocities and enhanced on-state currents compared to Si, which has also been experimentally verified [25, 31].

This article aims to systematically review 2D-material-based integrated electronics at the angstrom node, summarizing advances in material synthesis, device fabrication, and system integration, elucidating key physical mechanisms, and outlining future directions. The review is organized around core technical themes, with full chapters dedicated to the following topics: Wafer-Scale 2D Material Synthesis and Transfer Techniques, High-Performance 2D Transistor Fabrication for Advanced Nodes (including gate dielectric and source/drain contact engineering, as well as device performance in small-pitch architectures), 2D Circuits and Integrated Systems (including basic circuit functionalities, complex logic chips, memory chips, bioinspired chips, and three-dimensional integration strategies), and a final chapter offering an outlook on the scalable implementation of 2D materials at angstrom nodes from material, device, and system perspectives.

## WAFER-SCALE 2D MATERIAL SYNTHESIS AND TRANSFER TECHNIQUES

### Large-scale wafer-level synthesis of high-quality 2D materials

**Figure 2.**
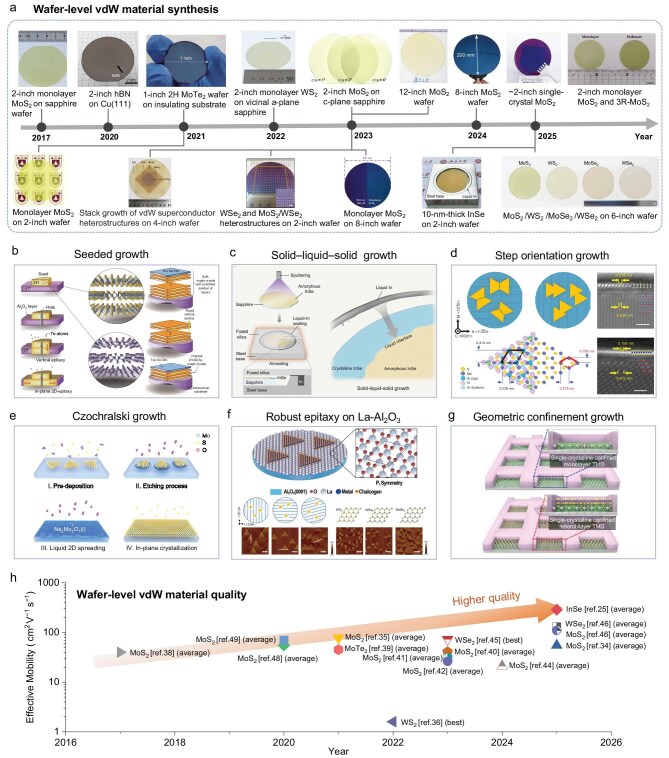
Recent progress in wafer-scale synthesis of 2D materials. (a) Research progress in wafer-level vdW material synthesis [25, 34–47]. hBN: hexagonal boron nitride. Copyright 2025, American Association for the Advancement of Science; Copyright 2025, Springer Nature; Copyright 2021, Springer Nature; Copyright 2021, Springer Nature; Copyright 2020, Springer Nature; Copyright 2017, American Chemical Society; Copyright 2021, American Association for the Advancement of Science; Copyright 2023, Springer Nature; Copyright 2023, Springer Nature; Copyright 2023, Springer Nature; Copyright 2025, Springer Nature; Copyright 2024, Springer Nature; Copyright 2023, Springer Nature; Copyright 2025, American Association for the Advancement of Science; Copyright 2023, Springer Nature. (b) Seed-assisted growth of a 1-inch single-crystalline 2H-MoTe_2_ wafer [39]. Copyright 2021, American Association for the Advancement of Science. (c) S–L–S conversion route enabling 2-inch high-quality InSe wafer synthesis [25]. Copyright 2025, American Association for the Advancement of Science. (d) Step-orientation-induced epitaxy for 2-inch monolayer MoS_2_ single-crystal growth on sapphire substrates [35]. Scale bars: 1 nm. Copyright 2021, Springer Nature. (e) 2D Czochralski (2D-CZ) growth method for 2-inch single-crystalline MoS_2_ wafer fabrication [34]. Copyright 2025, Springer Nature. (f) Lanthanum (La)-passivated epitaxy on sapphire substrates, achieving 6-inch single-crystal TMD films, including MoS_2_, MoSe_2_, WS_2_, and WSe_2_ [46]. Copyright 2025, American Association for the Advancement of Science. Scale bars (left to right): 1 μm, 0.2 μm, 0.6 μm, 0.3 μm, 0.3 μm and 0.6 μm. (g) Geometry-constrained selective growth strategy realizing wafer-scale arrays of single-domain monolayer WSe_2_ and single-domain MoS_2_/WSe_2_ heterostructures [45]. Copyright 2023, Springer Nature. (h) Comparison of experimentally measured room-temperature carrier mobilities in wafer-scale 2D materials [25, 34–36, 38–42, 44–46, 48, 49].

The realization of large-scale, ultra-short-channel electronic devices based on 2D materials fundamentally relies on the wafer-level synthesis of high-quality 2D semiconductor thin films. The quality of wafer-scale 2D materials is typically defined by the following key criteria: (i) precise layer-number control (that is, monolayer or few-layer uniformity) and wafer-scale uniformity in thickness and properties, (ii) single-crystalline or large single-domain size, (iii) low defect density (minimal vacancies, grain boundaries, and wrinkles), and (iv) high and spatially uniform carrier mobility, which is critical for transistor performance. The most widely adopted synthesis techniques to date include chemical vapor deposition (CVD), metal–organic chemical vapor deposition (MOCVD), and molecular beam epitaxy (MBE) [32, 33]. With continuous advances in these methods, significant progress has been made toward scalable epitaxy and monocrystalline growth of 2D materials. Figure 2a summarizes the evolution of wafer-scale 2D material synthesis since 2017 [25, 34–47]. Recent studies have demonstrated the successful epitaxial growth of 6-inch monocrystalline MoS_2_, WS_2_, MoSe_2_, and WSe_2_ films, marking a critical step toward the integration of 2D materials into next-generation semiconductor platforms [46].

Figure 2b–g illustrates six representative wafer-scale synthesis strategies for 2D materials, which are based on distinct underlying physical mechanisms. In Fig. 2b, Xu *et al.* [39] reported a novel approach for synthesizing wafer-scale, single-crystal semiconducting-phase molybdenum telluride (MoTe_2_) films through a combined phase-transformation and recrystallization process. This preparation method is achieved through the diffusion and rearrangement of atoms, without the need for a substrate as a template. Therefore, it can be carried out on amorphous SiO_2_ substrates, providing a foundation for subsequent device fabrication. In Fig. 2c, Qin *et al.* [25] reported a solid–liquid–solid (S–L–S) conversion strategy to realize wafer-scale, high-crystallinity, single-phase InSe thin films. By constructing an indium-rich liquid interface and maintaining an exact 1 : 1 In:Se stoichiometric ratio, the amorphous indium selenide precursor is transformed into phase-pure, highly crystalline InSe, exhibiting exceptional film uniformity and crystallographic integrity across a 5 cm wafer.

In Fig. 2d, Li *et al.* [35] demonstrated the epitaxial growth of 2-inch single-layer MoS_2_ monocrystals achieved on C-plane sapphire substrates with a deliberate miscut along the *A*-axis. This vicinal orientation effectively breaks the degeneracy of anti-parallel MoS_2_ domain nucleation energies, leading to unidirectional domain alignment exceeding 99% and enabling wafer-scale single-domain coverage. In Fig. 2e, Jiang *et al.* [34] reported a two-dimensional Czochralski (2D-CZ) growth technique, which introduces a controlled S–L–S phase transformation to convert polycrystalline MoS_2_ into large-area single crystals. Single-layer MoS_2_ synthesized via the 2D-CZ method exhibits a single-crystal domain size of 1.5 cm and a defect density of 2.9 × 10^12^ cm^−2^. As shown in Fig. 2f, Zou *et al.* [46] demonstrated that a lanthanum (La)-passivated sapphire substrate enables robust epitaxial growth of transition metal dichalcogenides (TMDs) by breaking the inversion symmetry of the sapphire surface through a single atomic La layer. This interfacial symmetry breaking promotes lattice alignment and suppresses rotational domain formation, resulting in the growth of 6-inch monocrystalline TMDs, including MoS_2_, MoSe_2_, WS_2_, and WSe_2_. In Fig. 2g, Kim *et al.* [45] reported a geometrically confined selective-area growth method, in which patterned SiO_2_ masks define micron-scale growth windows, effectively constraining nucleation sites. This approach yields wafer-scale arrays of single-domain monolayer WSe_2_, which can be further extended to form MoS_2_/WSe_2_ heterojunctions with precise registry, providing a viable route toward deterministic van der Waals (vdW) heterostructure integration.

Figure 2h summarizes the carrier mobilities of 2D materials synthesized using various wafer-scale growth techniques [25,34–36,38–42,44–46,48,49]. With continued advances in epitaxial quality and interfacial engineering, the field-effect effective mobility and spatial uniformity of 2D semiconductor thin films have improved substantially, highlighting their increasing suitability for short-channel field-effect transistors and prospective angstrom technology nodes. Overall, wafer-level synthesis of 2D materials is undergoing a transition from exploratory laboratory studies toward scalable, low-defect, industrial-grade manufacturing, thereby establishing a robust materials foundation for post-silicon integrated electronics.

### Wafer-scale 2D material transfer and stacking

**Figure 3.**
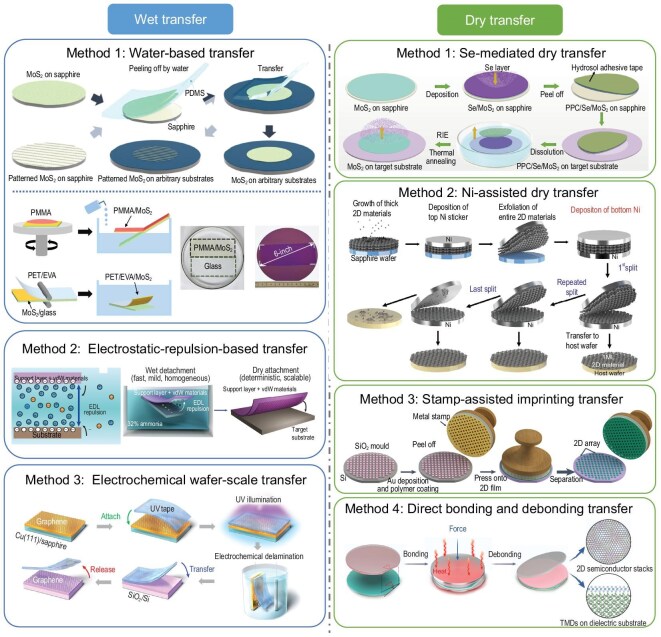
Wet transfer methods for wafer-level 2D materials [38,50–52] and dry transfer methods [53–56]. PDMS: polydimethylsiloxane; PMMA: polymethyl methacrylate; PET: polyethylene terephthalate; EVA: ethylene vinyl acetate; EDL: electric double layer; PPC: polycarbonate; RIE: reactive ion etching. Copyright 2017, American Chemical Society; Copyright 2018, Springer Nature; Copyright 2025, Springer Nature; Copyright 2024, Springer Nature; Copyright 2025, Springer Nature; Copyright 2025, American Association for the Advancement of Science; Copyright 2025, Springer Nature.

Despite substantial progress in wafer-scale epitaxial growth of 2D materials, their integration into advanced electronic device fabrication still critically depends on high-precision transfer and stacking technologies. Since 2D materials are often grown on heterogeneous substrates such as sapphire, SiO_2_/Si, or glass, transferring them onto process-compatible silicon wafers or interconnect platforms is essential for practical device integration. Current wafer-scale transfer strategies are generally categorized into wet and dry approaches, as shown in Fig. 3, each offering distinct advantages in cleanliness, controllability, and scalability.

Wet transfer techniques typically employ liquid-mediated peeling and reattachment. Representative strategies include: (i) utilizing surface tension and capillary forces of water to achieve smooth, low-damage transfer between interfaces [38, 50]; (ii) employing the electrostatic repulsion of an electric double layer formed in ammonia solution to enable rapid, clean, and etchant-free separation of 2D films [51], and (iii) adopting UV-sensitive adhesive tapes (UV tapes) with controllable adhesion under ultraviolet illumination and electrochemical delamination to achieve large-area, damage-free wafer-scale transfer [52].

Dry transfer methods, in contrast, avoid chemical contamination and enable superior interface control. Typical approaches include: (i) selenium (Se)-mediated intermediate-layer transfer for wafer-scale monolayer MoS_2_ with atomically clean and structurally intact interfaces [53]; (ii) a universal layer-resolved splitting process, in which multilayer 2D materials are first grown and subsequently separated stepwise into monolayers for wafer-scale transfer [54]; (iii) metal stamp-assisted micro-imprinting for selective patterning and transfer of high-quality 2D material arrays without introducing polymer or chemical residues [55]; and (iv) direct wafer bonding and debonding processes to fabricate 2D semiconductor stacks with engineered layer numbers and controlled interlayer twist angles [56].

In summary, as device scaling approaches the angstrom regime, stringent requirements on interfacial cleanliness, layer uniformity, and defect-free stacking are emerging. The maturity of wafer-scale transfer and stacking technologies will thus play a decisive role in enabling the large-scale commercialization of 2D materials for next-generation logic, heterogeneous integration, and three-dimensional chip architectures.

## HIGH-PERFORMANCE FIELD-EFFECT TRANSISTORS BASED ON 2D MATERIALS

As of 2024, silicon-based devices have entered the mass production stage of the 3-nanometer node, and the IRDS indicates that the angstrom node will become the core target of semiconductor technology after 2030 [12]. However, as device dimensions approach the angstrom scale, the limitations of traditional silicon-based materials have become increasingly prominent: first, the short-channel effect intensifies sharply, and electron mobility decreases significantly due to quantum confinement effects; second, the silicon surface contains a large number of dangling bonds, leading to higher density of interface states between the gate dielectric and the channel, resulting in degraded gate control capability; third, performance degradation caused by the miniaturization of contact electrodes.

Against this background, 2D semiconductor materials (such as MoS_2_, WSe_2_, Bi_2_O_2_Se, etc.) have demonstrated irreplaceable advantages due to their unique layered crystal structures [11]. 2D semiconductors feature atomically uniform thickness (as low as 0.6–1.2 nm), dangling bond-free surfaces, and excellent chemical stability, which can effectively suppress the short-channel effect.

Nevertheless, 2D integrated electronics for angstrom nodes still face multiple challenges: interface matching between high-*κ* gate dielectrics and 2D semiconductors, Fermi-level pinning-free low contact resistance, high ballistic rate channels, process compatibility of novel 3D device structures, and integration with existing silicon-based complementary metal oxide semiconductor (CMOS) foundries. This section systematically elaborates on the technical breakthroughs and future directions of 2D integrated electronics from four dimensions: gate dielectric preparation, source-drain contact engineering, transistor size miniaturization, and advanced structure transistors, aiming to provide a comprehensive reference for the development of angstrom node devices.

### Gate dielectric preparation: high-κ integration and interface regulation

As a core component of FETs, the dielectric constant (*κ*), interface quality, and physical thickness of the gate dielectric directly determine the device’s gate control capability and leakage current characteristics. For angstrom node devices, to meet the requirement of EOT ($\frac{{{t}_{{\mathrm{ox}}}{\varepsilon }_{{\mathrm{Si}}{{\mathrm{O}}}_2}}}{{{\varepsilon }_{{\mathrm{ox}}}}}$, where *t*_ox_ is the physical thickness of the gate dielectric, ${\varepsilon }_{{\mathrm{Si}}{{\mathrm{O}}}_2}$ is the dielectric constant of SiO_2_, and *ε*_ox_ is the dielectric constant of target high-*κ* gate dielectric) <0.5 nm, traditional SiO_2_ dielectrics with low *κ* value (≈ 3.9) present a sharp increase in quantum tunneling leakage current [57]. Therefore, the integration of high-*κ* dielectrics (such as HfO_2_, Al_2_O_3_, SrTiO_3_, etc.) has become an inevitable choice. However, although the dangling bond-free surface of 2D semiconductors can suppress interface states, it also makes it difficult for high-*κ* dielectrics to form uniform and dense thin films through traditional deposition methods—this ‘high-*κ* dielectric deposition challenge’ is a key bottleneck restricting the performance of 2D devices.

The deposition of high-*κ* dielectric materials presents several core challenges that must be addressed to fully exploit their potential in advanced 2D transistors. The surface of 2D semiconductors (such as MoS_2_, graphene) is a vdW interface with weak interatomic forces and no active groups. This causes high-*κ* precursors (such as Hf[N(CH_3_)_2_]_4_) hard to adsorb and decompose on their surfaces, easily forming island-like growth (Volmer–Weber mode), resulting in discontinuous dielectric films and low coverage [58]. In addition, the interlayer forces of 2D semiconductors are weak. High temperatures (>200°C) or plasma treatments in traditional atomic layer deposition (ALD) processes may lead to interlayer peeling or lattice damage of 2D semiconductor materials, deteriorating interface quality. Therefore, the development of high-*κ* dielectric deposition strategies suitable for 2D semiconductors needs to address both the ‘nucleation difficulty’ and ‘interface damage’ issues.

The integration of high-*κ* dielectric materials into semiconductor devices can be pursued through several distinct technical paths. To address the challenges of difficult nucleation and poor interface quality of high-*κ* dielectrics caused by the dangling bond-free surface of 2D semiconductors, current research has formed two mainstream technical paths—the vdW heterointegration strategy and the ALD with seed layer method [59, 60]. Through precise regulation of interface interactions and nucleation mechanisms, efficient compatibility between high-*κ* dielectrics and 2D semiconductors is achieved.

Damage-free transfer techniques have emerged as a pivotal approach for achieving effective interface regulation in vdW heterointegration, thereby preserving the intrinsic properties of 2D materials. The core idea is to first prepare high-quality high-*κ* dielectric films on compatible substrates (such as mica and sapphire), then transfer them to the surface of 2D semiconductors without damage through vdW transfer technology. The key advantages of this path lie in the ability to pre-optimize the crystal quality and thickness uniformity of high-*κ* dielectrics while maximizing the retention of the intrinsic electrical properties of 2D semiconductors. This section focuses on two core directions: transferred perovskite dielectrics and transferred Al_2_O_3_ dielectrics [61, 62].

**Figure 4.**
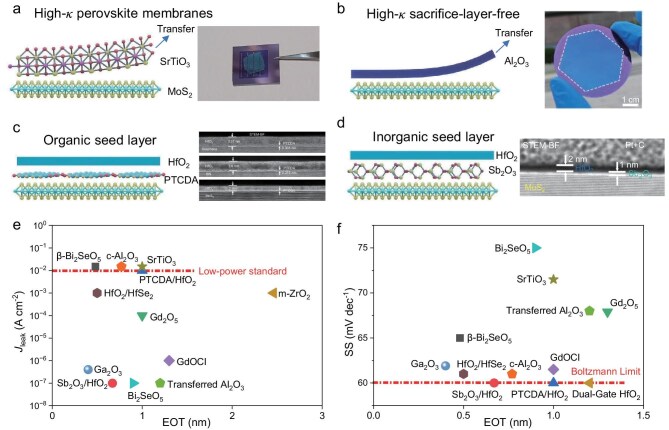
Schematic diagrams of integration methods and electrical properties of 2D gate dielectrics. (a) Large-area vdW transfer of ultra-high-*κ* gate dielectric (SrTiO_3_) [61]. Copyright 2022, Springer Nature. (b) Damage-free large-area transfer of high-*κ* gate dielectrics (Al_2_O_3_) without a sacrifice layer [62]. Copyright 2025, Springer Nature. (c) Organic molecular crystal (PTCDA) seed layer provides nucleation sites for ALD of gate dielectric (HfO_2_), enabling ultra-thin vdW gap [64]. Copyright 2019, Springer Nature. (d) Inorganic molecular crystal (Sb_2_O_3_) seed layer provides nucleation sites for ALD of gate dielectric (HfO_2_), achieving ultra-thin EOT [65]. STEM-BF: scanning transmission electron microscopy bright-field. Copyright 2023, Springer Nature. (e) The correlation between EOT and *J*_leak_ of high-*κ* dielectrics compatible with 2D semiconductors. (f) Relationship between the EOT of 2D-compatible high-*κ* dielectrics and the SS of 2D transistors [57, 61, 64–75].

Perovskite oxides (such as SrTiO_3_) have become ideal choices for high-*κ* dielectrics in 2D devices due to their ultra-high dielectric constant (bulk *κ* ≈ 300), atomically flat surfaces, and excellent chemical stability [63]. Huang *et al.* developed transferable ultra-high-*κ* single-crystal SrTiO_3_ films as gate dielectrics for 2D field-effect transistors, as shown in Fig. 4a. The SrTiO_3_ they developed exhibits an ideal capacitance equivalent thickness (CET) (<1 nm) and low leakage current (<10^−2^ A/cm^2^). After transfer, a vdW gap of ∼0.3 nm is formed between SrTiO_3_ and the 2D semiconductor, which can effectively suppress carrier tunneling leakage current and avoid the generation of interface states caused by chemical bonding. The fabricated MoS_2_ short-channel transistor achieves an SS of ∼70 mV/dec and an on/off ratio as high as 10^7^ [61].

Due to its stable dielectric constant (*κ* ≈ 9), high chemical inertness, and mature ALD process, Al_2_O_3_ is one of the earliest high-*κ* dielectrics to achieve transfer integration. The core technical challenge lies in avoiding film cracking and interface contamination during the transfer process. He *et al.* developed a damage-free transfer method using water. A natural vdW gap of ∼0.5 nm exists between mica and Al_2_O_3_. The transferred Al_2_O_3_ film has a surface roughness (*R*_a_) as low as 0.12 nm, and the EOT of a 2.8 nm-thick transferred Al_2_O_3_ reaches 1.86 nm. This technology has achieved complete transfer of 2.2 cm hexagonal Al_2_O_3_ wafers with a transfer yield of 100%, as shown in Fig. 4b, providing a wafer-level solution for high-density integration of 2D devices [62].

Besides, the combination of ALD with a seed layer enables the precise construction of nucleation sites, which is critical for achieving high-quality thin films. ALD technology is a mainstream process for high-*κ* dielectric integration due to its high film thickness controllability (precision of 0.1 nm) and good coverage. However, the lack of active nucleation sites on the surface of 2D semiconductors makes it difficult for ALD precursors to adsorb. The introduction of seed layers can solve the ALD nucleation problem by constructing artificial active sites. Two commonly employed seed-layer materials for ALD on 2D semiconductors are the organic molecular crystal PTCDA and the inorganic molecular crystal Sb_2_O_3_, which provide artificial nucleation sites to facilitate uniform dielectric growth [64, 65].

The carbonyl groups (C=O) in 3,4,9,10-perylenetetracarboxylic dianhydride (PTCDA) molecules can serve as adsorption sites for ALD precursors, and form vdW contacts with 2D semiconductors through π–π interactions to avoid chemical damage. Li *et al.* developed a monolayer PTCDA seed layer with a vdW gap of ∼0.31 nm between it and the 2D semiconductor, as shown in Fig. 4c. Using PTCDA as the seed layer, HfO_2_ was grown by ALD at 150°C, and a continuous film with a thickness of 1.45 nm could be formed in only 7 cycles, with an EOT as low as 1 nm. The MoS_2_ FET achieved an SS of 60 mV/dec. The PTCDA seed layer is also compatible with various 2D materials such as BN and WSe_2_, and the transfer yield on wafer-level CVD MoS_2_ reaches 90% [64].

As an inorganic molecular crystal, Sb_2_O_3_ has the advantages of high dielectric constant (*κ* ≈ 11.5) and strong vdW interactions with 2D semiconductors. Its core function is to construct a hydrophilic interface by covering the hydrophobic regions on the surface of 2D semiconductors, which can efficiently capture H_2_O precursors during ALD, reducing the nucleation of HfO_2_ from 15 cycles to 3 cycles. Xu *et al.* reported performance breakthroughs of HfO_2_/Sb_2_O_3_ hybrid dielectrics: the hybrid dielectric of 1 nm Sb_2_O_3_ + 2 nm HfO_2_ achieves an EOT as low as 0.67 nm, as shown in Fig. 4d. The monolayer MoS_2_ FET based on this dielectric achieves an on/off ratio exceeding 10^6^ and an SS of 62.8 mV/dec at an ultra-low operating voltage of 0.4 V. This technology can also be extended to other high-*κ* dielectrics such as ZrO_2_ and Al_2_O_3_ [65].

To evaluate the integration effect of high-*κ* dielectrics, three key indicators need to be focused on: EOT, leakage current density (*J*_leak_), and SS, in addition to critical breakdown electric field (*E*_m_) and interface state density (*D*_it_). Figure 4e shows the relationship between EOT and *J*_leak_ of high-*κ* dielectrics matching 2D semiconductor materials, as well as the gap relative to the leakage current allowed by current silicon-based transistors (10^−2^ A/cm^2^). Figure 4f shows the relationship between EOT of high-*κ* dielectrics matching 2D semiconductor materials and the SS of 2D transistors, as well as the gap with the room-temperature Boltzmann limit (60 mV/dec) [57, 61, 64–75].

In the future, for angstrom nodes, gate dielectric preparation needs to further break through the balance between ‘EOT reduction’ and ‘leakage current suppression’: on the one hand, through the stack design of high-*κ*/ultra-high-*κ* dielectrics (such as LaAlO_3_, Ta_2_O_5_), continue to reduce EOT while minimizing *J*_leak_; on the other hand, develop atomically precise interface modification technologies (such as single-atomic-layer doping) to reduce *D*_it_ to below 10^10^ cm^−2^ eV^−1^, laying the foundation for device performance optimization [76]. Furthermore, atomic-scale transmission electron microscopy (TEM) characterization facilitates direct visualization of the dielectric–2D material interface quality, which is critical for validating the efficacy of interface regulation strategies [77].

### Source-drain contact engineering: ohmic contact and carrier transport regulation

Source-drain contacts are critical interfaces for carriers to inject from metal electrodes into the channel in 2D FETs, and their contact resistance (*R*_c_) directly affects the device’s on-state current and switching speed. For advanced node devices, the transport mode is ballistic transport, so the proportion of *R*_c_ in the total resistance is greatly increased [78]. However, the contact between 2D semiconductors and metal electrodes generally suffers from the Fermi-level pinning effect—the Fermi level (*E*_f_) of the metal is pinned inside the bandgap of the 2D semiconductor, forming a high Schottky barrier (*Φ*_SB_), which makes carrier injection difficult and increases *R*_c_ [79]. Therefore, eliminating Fermi-level pinning and achieving low-resistance ohmic contacts are the core goals of source-drain contact engineering.

The performance and reliability of semiconductor devices are critically influenced by the Fermi-level pinning effect, making the study of its origins and accurate characterization essential for material and interface engineering [80]. When a metal contacts a 2D semiconductor, defect states or dangling bonds are formed at the interface, causing the Fermi level not to change with temperature and doping. Unlike silicon semiconductor systems, the electronic structure of 2D semiconductors is extremely sensitive to interface interactions: when a metal (such as Ti) is in direct contact with a 2D semiconductor (such as MoS_2_), the metal atoms form chemical bonds (such as Ti–S bonds) with the atoms on the surface of the 2D material, leading to an increase in the density of defect states in the 2D semiconductor and *E*_f_ being pinned in the middle of the bandgap; when the metal is combined with the 2D semiconductor through weak vdW forces (no chemical bonds), the density of defect states is low. However, vdW contacts result in insufficient overlap between the atomic orbitals of the metal and the 2D material, leading to low efficiency of carrier transfer from the metal electrode to the 2D material and thus high contact resistance [81].

Due to the different band structures of n-type and p-type 2D semiconductors, the implementation strategies for their ohmic contacts need to be designed specifically. The core principle is to regulate the energy band on the semiconductor surface so that the Fermi level enters the conduction band or valence band. Current research mainly adopts four methods: semi-metal contact, doping regulation, interface intercalation, and vdW epitaxy.

#### Semi-metal Bi for n-type ohmic contact

**Figure 5.**
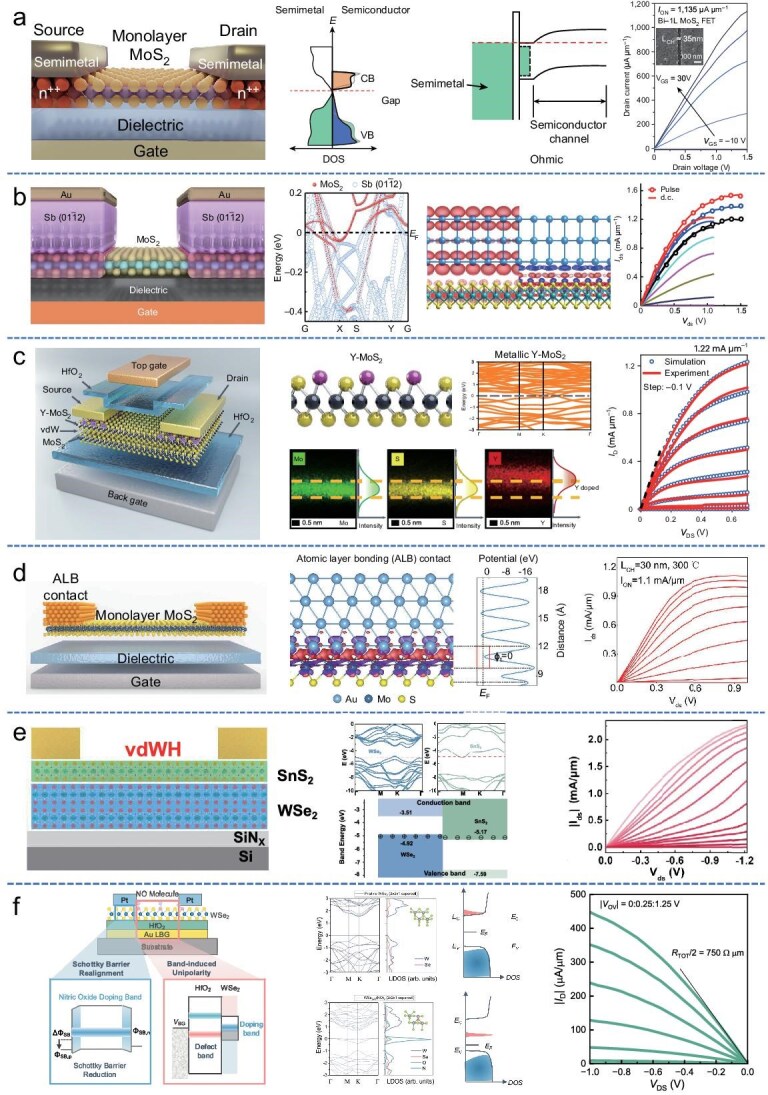
Schematic illustration of the device structures, operating principles, and performance characterization methodologies for Ohmic contacts in 2D transistors. (a) Cross-sectional view, density of states distribution diagram, band structure diagram, and output characteristic curve of Bi-MoS_2_ contact transistor [82]. DOS: density of states; CB: conduction band; VB: valence band. Copyright 2021, Springer Nature. (b) Cross-sectional view, band structure diagram, atomic arrangement diagram, and output characteristic curve of Sb-MoS_2_ contact transistor [84]. Copyright 2023, Springer Nature. (c) Cross-sectional view, band structure diagram, TEM image, and output characteristic curve of Y-MoS_2_ contact transistor [85]. Copyright 2024, Springer Nature. (d) Cross-sectional view, atomic arrangement diagram, and output characteristic curve of atomic layer bonding contact transistor [86]. Copyright 2025, American Association for the Advancement of Science. (e) Cross-sectional view of 2D FET, band structure diagrams of WSe_2_ and SnS_2_, and output characteristic curves for 20 nm channel lengths [87]. vdWH: van der Waals heterostructure. Copyright 2025, American Association for the Advancement of Science. (f) Cross-sectional view, band structure diagram, and output characteristic curve of Pt-WSe_2_ (NO) contact transistor [89]. LDOS: local density of states. Copyright 2025, Springer Nature.

Utilizing the semi-metallic properties of Bi (near-zero density of states at the Fermi level) to form contacts with 2D semiconductors (such as MoS_2_ and WS_2_), since the density of states at the Fermi level of Bi is small, the metal-induced gap states (MIGS) introduced by it on the surface of 2D semiconductors can be almost ignored, thereby suppressing Fermi-level pinning. Meanwhile, The Fermi level of Bi is higher than the bottom of MoS_2_ conduction band, achieving barrier-free ohmic contact (lowest contact resistance as low as 123 Ω μm at a carrier density (*n*_2D_) of 1.5 × 10^13^ cm^−2^), and the on-state current density of the MoS_2_ FET reaches 1135 μA/μm, as shown in Fig. 5a. Moreover, the Bi (0001) plane is arranged parallel to the MoS_2_ plane, ensuring atomic-level flatness of the interface and reducing carrier scattering. In terms of compatibility, it is suitable for various 2D semiconductors such as MoS_2_, WS_2_, and WSe_2_ [82].

#### Semi-metal Sb for n-type ohmic contact

By regulating the crystal orientation of the Sb (0112) plane, strong vdW interaction-mediated band hybridization is formed with 2D semiconductors (such as MoS_2_), breaking through the charge transport limit of traditional vdW gaps. When the semi-metal Sb contacts MoS_2_ in a specific crystal orientation, hybrid coupling of surface electronic orbitals occurs. The hybridization shifts the conduction band bottom of MoS_2_ below the Fermi level of Sb, and an n-type degenerate doping is formed in the contact region on the semiconductor surface, achieving ohmic contact (lowest contact resistance as low as 42 Ω μm at a carrier density (*n*_2D_) of 3 × 10^13^ cm^−2^), and the on-state current density of the MoS_2_ FET reaches 1.23 mA/μm, as shown in Fig. 5b. At the same time, the vdW gap at the interface is reduced to 0.285 nm, which is smaller than the intrinsic MoS_2_ interlayer spacing (0.615 nm) [83] and the Sb (0112) lattice spacing (0.315 nm), enhancing charge injection [84]. Compared with Bi, the semi-metal Sb has stronger thermal stability. The melting point of Sb (>600°C) is much higher than that of Bi, and the (0112) oriented structure is thermodynamically stable, with no performance degradation after 24 h at 125°C, which to a certain extent makes up for the thermal stability defect of Bi contacts.

#### Y doping-induced phase transition for n-type ohmic contact

For MoS_2_ semiconductors, substituting surface S atoms with Y atoms induces a phase transition of MoS_2_ from a semiconductor phase (2H phase) to a zero-bandgap metallic phase (Y-MoS_2_), forming a vdW metal buffer layer. This phase transition converts the original Schottky barrier and Fermi-level pinned metal-semiconductor contact into a metal–metal-like contact; meanwhile, the Fermi level of Y-MoS_2_ is shifted up to align with the conduction band of the channel 2H-MoS_2_ through Y doping. The synergistic effect of phase transition and degenerate doping achieves an ohmic contact resistance (average contact resistance as low as 69 Ω μm and lowest contact resistance as low as 43 Ω μm), which is much better than traditional Ti/Au contacts, as shown in Fig. 5c [85].

#### Atomic layer bonding for n-type contact

This technology achieves an ultra-low contact resistance (as low as 70 Ω μm at a carrier density (*n*_2D_) of 2.7 × 10^13^ cm^−2^) and high-temperature stability up to 400°C (aligning with CMOS BEOL thermal budgets) by precisely removing the upper-layer sulfur atoms of 2D TMDs, taking monolayer MoS_2_ as an example, enabling the formation of a metal coherent bonding interface between the transition metal atomic layer and the metal electrode, as shown in Fig. 5d [86].

#### vdW heterojunction intercalation and gate band modulation for p-type ohmic contact

1L-SnS_2_/2L-WSe_2_ vdW heterojunctions were prepared by CVD, which form a Type-III broken band structure—the valence band maximum (−4.92 eV) of 2L-WSe₂ is higher than the conduction band minimum (−5.17 eV) of 1L-SnS₂. This band alignment enables electrons to spontaneously tunnel from the valence band of WSe_2_ to the conduction band of SnS_2_, achieving p-type doping of WSe_2_. There is no chemical bonding at the heterojunction interface, avoiding lattice defects and carrier scattering. When a negative gate voltage is applied, the upward shift of the valence band of WSe_2_ (close to the gate) is greater than that of the conduction band of SnS_2_ (far from the gate), increasing the band breaking difference, further enhancing interlayer charge transfer, and significantly increasing the hole density. Finally, an ultra-high 2D hole density of 1.49 × 10^14^ cm^−2^ is achieved, which is much higher than the traditional electrostatic doping limit (<3 × 10^13^ cm^−2^). The ultra-high hole density forms a strong degenerate state in the WSe_2_ contact region, with a lowest contact resistance as low as 41 Ω μm. The on-state current density of the 20 nm channel device reaches 2.30 mA/μm, the highest value reported for 2D transistors to date, as shown in Fig. 5e. In terms of uniformity: 1.5 × 1.5 cm^2^ large-area SnS_2_/WSe_2_ heterostructured films yield 130 devices with average *I*_on​_ > 1.0 mA/μm and switching ratio of 10^10^ [87].

#### VdW heterojunction epitaxial contact

Researchers epitaxially grew VSe_2_ nanosheets on the surface of CVD-grown bilayer WSe_2_ nanosheets. Utilizing the difference in thermal expansion coefficients between VSe_2_ and WSe_2_, the gap between VSe_2_ domains is regulated by adjusting the growth temperature, which serves as the channel length of the 2D transistor. VSe_2_ exhibits excellent contact performance with WSe_2_, achieving an on-state current density of 1.72 mA/μm at a channel length of 20 nm (lowest on-state resistance as low as 500 Ω μm) [88].

#### NO doping-induced doping band for p-type near-ohmic contact

For WSe_2_, the combination of NO molecules with Se vacancies on the semiconductor surface forms a doping band in the bandgap. The Fermi level moves toward the valence band, achieving Schottky barrier rearrangement and p-type near-ohmic contact resistance (lowest contact resistance as low as 390 Ω μm at a carrier density (*n*_2D_) of 1.22 × 10^13^ cm^−2^), as shown in Fig. 5f. While reducing the hole Schottky barrier, the electron barrier is increased, realizing p-type unipolar transport. Devices maintain stable performance for 24 days under moderate thermal conditions, with no significant degradation in key metrics [89].

#### Band-hybridized selenium contact for p-type semiconductors

The ultrathin Se interfacial layer (highest work function of 5.9 eV) forms a semimetallic contact with Au by band hybridization, suppressing MIGS and reducing Schottky barrier height. Applied to WSe_2_ FETs, it achieves a low contact resistance of 540 Ω μm and a high saturated on-state current density of 430 μA/μm (80-nm channel). This scalable method is transferable to black phosphorus and carbon nanotubes, enabling reliable low-resistance contacts for p-type nanodevices. In terms of reliability, annealing at 200°C in inert gas results in negligible changes to transfer characteristics; X-ray photoelectron spectroscopy (XPS) confirms stable Se presence at the contact interface post-annealing [90]. A benchmark of state-of-the-art high-performance 2D FETs is provided in Table 1, which compiles key device metrics including channel length, gate dielectric thickness, contact strategy, on-state current, and subthreshold swing across leading research institutions.

**Table 1.** Benchmark of high-performance 2D FETs.

Institution	Type	Material	*L*_ch_ (nm)	Gate dielectric (nm)	Contact strategy	*I*_on_ (μA/μm)	SS (mV/dec)	Ref.
**MIT**	n	1L-MoS_2_	35	SiN_*x*_/100	Bi	1135	–	[82]
**NJU**	n	1L-MoS_2_	20	HfO_2_	Sb	1230	180	[84]
**NJU**	n	3L-MoS_2_	20	HfO_2_/3	MBE-Sb	1080	76	[91]
**NJU**	n	1L-WS_2_	10	HfO_2_/10	Sb	675	130–140	[92]
**FDU**	n	1L-MoS_2_	30	HfO_2_/7	Au/Ti/Ni	300	–	[93]
**USTB**	n	1L-MoS_2_	30	HfO_2_/10	ALB-Au	1000	100	[86]
**Samsung**	n	2L-MoS_2_	30	HfO_*x*_/10	Au	1550	–	[94]
**PKU**	n	3L-MoS_2_	10	HfO_2_/2.6	Y-doped	1220	78	[85]
**PKU**	n	3L-InSe	20	HfO_2_/2.6	Y-doped	1350	69	[78]
**HNU**	p	2L-WSe_2_	20	SiN_*x*_/70	1L-SnS_2_	2300	–	[87]
**HNU**	p	2L-WSe_2_	20	SiN_*x*_/70	VSe_2_	1720	–	[88]
**PolyU**	p	3L-WSe_2_	80	HfO_2_/20	Au/Se	430	–	[90]
**PU**	p	NO-doped 2L-WSe_2_	55	HfO_2_/3.5	Ti/Pt	448	185	[89]
**PSU**	p	NO-doped 2L-WSe_2_	100	SiO_2_/285	Pd/Pt	421	350	[95]
**WHU**	p	2L-MoTe_2_	130	Al_2_O_3_	m-Te	124	–	[96]

Note that some devices in the reference lack information of gate dielectric thickness and subthreshold swing.

Scalability and CMOS compatibility are pivotal for 2D device industrialization. Most low-resistance contact strategies (for example, Sb (0112) vdW contact, ALB contact) adopt electron beam thermal evaporation and plasma etching—processes fully compatible with mainstream CMOS equipment. Their low thermal budget (<400°C) matches BEOL thermal thresholds, avoiding conflicts with backend workflows. Future efforts should focus on wafer-scale uniform contact deposition, alignment accuracy optimization, and process module standardization, to translate single-device performance to high-yield integrated circuits.

In the future, for angstrom nodes, source-drain contact engineering needs to further focus on two directions: first, develop a universal ohmic contact scheme to achieve low-resistance contact between the same metal and n/p-type 2D semiconductors, laying the foundation for CMOS integration; second, through atomic-level interface regulation (such as single-atomic-layer semi-metal intercalation), reduce contact resistance to below 50 Ω μm while ensuring long-term contact stability (>10^4^ h) to meet industrialization requirements.

### Device size miniaturization: the potential of 2D semiconductors in miniaturization

The area miniaturization of semiconductor devices requires the simultaneous multi-dimensional miniaturization of channel length (*L*_ch_), contact length (*L*_c_), and gate pitch (Pitch). After the sub-3-nm node, silicon-based semiconductor systems face bottlenecks such as intensified short-channel effects, increased proportion of contact resistance, and uncontrollable parasitic capacitance. For example, when the *L*_ch_ of silicon-based FinFETs is reduced to below 10 nm, the drain-induced barrier lowering (DIBL) value exceeds 100 mV/V, and the SS degrades to above 100 mV/dec, completely deviating from the requirements of high-performance devices [97].

In contrast, the atomic-level thickness of 2D semiconductors makes them naturally suitable for full-scale miniaturization: first, the dangling bond-free surface can suppress interface states, and the surface lattice vibration scattering is weaker than that of traditional silicon materials, resulting in lower channel resistance and weakened short-channel effects; second, vdW interlayer interactions support vertical stacked three-dimensional integration, breaking through the Pitch limit of planar processes, and the layered structure allows atomic-level precise regulation of channels, contacts, and gates. This section discusses three parameters: contact length, pitch length, and channel length.

### Small-pitch transistors

**Figure 6.**
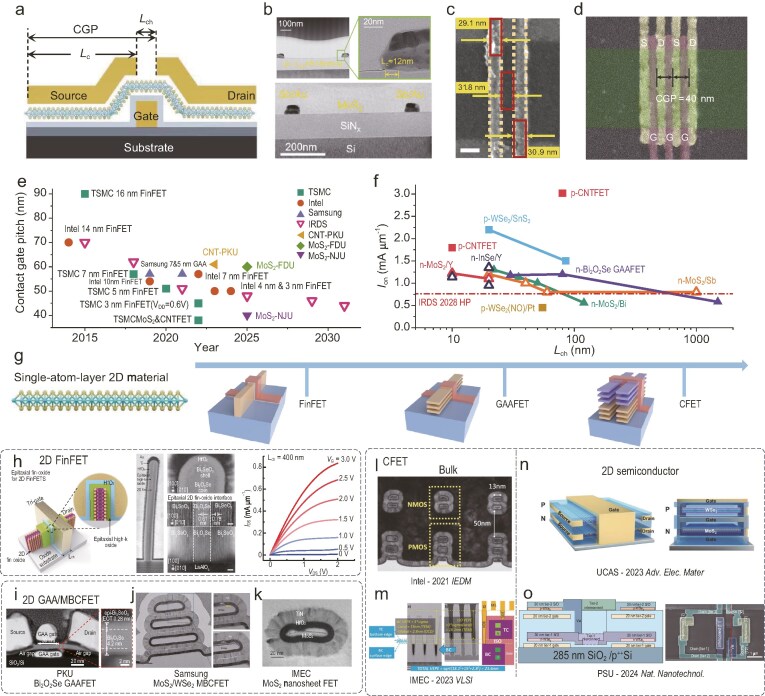
Transistor size miniaturization and architecture optimization. (a) Cross-sectional view of the transistor, showing the relationship between CGP, *L*_c_, and *L*_ch_. (b) TEM image of a 2D transistor with an ultra-short contact length (*L*_c_ ≈ 12 nm) [98,99]. Copyright 2023, IEEE; Copyright 2023, IEEE. (c) Scanning electron microscopy (SEM) image of a 2D transistor with 60 nm CGP [93]. Copyright 2025, Springer Nature. (d) SEM image of a 2D transistor with 40 nm CGP [91]. Copyright 2025, Springer Nature. (e) Benchmarks for the development of CGP from 2015 to 2030 by enterprises, research institutions, and IRDS, respectively [100–107]. (f) The relationship between channel length (*L*_ch_) and on-state current (*I*_on_) of representative low-dimensional materials transistors in the past 5 years [75,78,82,84,85,87,89,108,109]. (g) Simplified conceptual diagrams of the structures of FinFET, GAAFET and CFET. (h) Schematic structure diagram, SEM image of Fin, and output characteristic curve of 2D Bi_2_O_2_Se FinFET [110]. Copyright 2023, Springer Nature. (i) SEM images of 2D Bi_2_O_2_Se GAAFET and Bi_2_O_2_Se channel [109]. Copyright 2025, Springer Nature. (j) SEM image of 2D MoS_2_/WSe_2_ MBCFET [111]. Copyright 2024, IEEE. (k) SEM image and three-dimensional simulation diagram of 2D MoS_2_/WS_2_ Nanosheet FET [112]. Copyright 2024, IEEE. (l) SEM image of Intel Si-CFET [113]. Copyright 2021, IEEE. (m) SEM image and MOL interconnection schematic diagram of IMEC Si-CFET [114]. Copyright 2023, IEEE. (n) Schematic structure diagram of n-MoS_2_ and p-WSe_2_ CFET [115]. Copyright 2022, John Wiley and Sons. (o) Vertical structure schematic diagram and SEM image of n-WSe_2_ and p-WSe_2_ CFET [116]. Copyright 2024, Springer Nature.

To meet the requirements of transistor integration density, full-scale miniaturization also includes the reduction of contact length (*L*_c_) and contact-gate pitch (CGP) length. The relationship between CGP, *L*_c_, and *L*_ch_ of the device is shown in Fig. 6a. With the development of silicon-based integrated circuit technology, the channel length has approached the physical limit. Therefore, in the post-Moore era, the more important factor affecting integration density is the contact length. The reduction of contact length *L*_c_ directly affects device performance. The reduction in the area of the contact perpendicular to the current path direction will lead to an increase in contact resistance. Therefore, how to balance the increase in contact resistance and the increase in integration density has become a new problem. Wu *et al.* developed MoS_2_ transistors with an ultra-short contact length of 12 nm, as shown in Fig. 6b [98, 99]. Chen *et al.* reported high-performance MoS_2_ transistors with a pitch length of 60 nm (where *L*_c_ = 30 nm, *L*_ch_ = 30 nm), as shown in Fig. 6c. In addition to improving integration density, the miniaturization of CGP also reduces the overlap of interconnect metals, effectively reducing parasitic capacitance, lowering transmission delay, and reducing the dynamic power consumption of the circuit [93]. Du *et al.* reported high-performance MoS_2_ transistors with a Pitch length of 39 nm (where *L*_c_ = 18 nm, *L*_ch_ = 17 nm) by using ultra-high-vacuum MBE, as shown in Fig. 6d [91]. We have collected the development trends of CGP over time from enterprises, research institutions, and IRDS, as shown in Fig. 6e [100–107].

For 2D semiconductors, the miniaturization of channel size has inherent advantages: they have a wider bandgap than silicon, which means 2D transistors have smaller off-state current and lower static power consumption. In terms of on-state current, representative works of small-size, high-performance devices in the past 5 years are shown in Fig. 6f [75,78,82,84,85,87,89,108,109]. 2D short-channel high-performance transistors not only meet the requirements of integration density but also meet the IRDS 2028 requirements for high-performance transistors (780 μA/μm) [12].

### Advanced node 2D transistors: structural innovation and high-density integration

2D transistors need to leverage the advantage of atomic-level thickness to achieve leapfrog miniaturization of pitch through three-dimensional stacking, gate-all-around (GAA), and multi-bridge channel (MBC). As device dimensions approach the angstrom node, the short-channel effects, parasitic capacitance, and resistance issues of traditional planar FETs have become increasingly severe, making it difficult to meet performance and integration density requirements. Through novel structures such as GAA, MBC, and CFETs, the coordinated optimization of size reduction, performance improvement, and high-density integration is achieved, as shown in Fig. 6g.

2D materials hold distinct advantages over bulk silicon in advanced architectures such as GAA and CFET, especially for sub-3 nm scaling. Their atomic-level thickness (<3 nm) aligns with the ultra-thin channel demand of these structures, while inherently lower surface roughness minimizes scattering and boosts carrier mobility. Unlike bulk Si, which suffers severe mobility degradation when thinned to a few nanometers, 2D materials retain high mobility due to their dangling-bond-free surfaces. Endowed with atomic-scale thickness, dangling bond-free surfaces, vdW characteristics, and wider bandgaps, 2D materials offer irreplaceable advantages for three-dimensional transistors (GAA/MBC/CFET), realizing higher performance and lower leakage current.

#### Fin field-effect transistors

Fin field-effect transistor (FinFET) is constructed based on the epitaxial heterointegration of 2D semiconductor Bi_2_O_2_Se and native high-*κ* dielectric Bi_2_SeO_5_. The dangling bonds at the edges of the [Bi_2_O_2_]^2+^ layers form strong bonding with the substrate, driving vertical anisotropic growth to form Fin structures with a high aspect ratio (up to 20); then, the Se layers of the Bi_2_O_2_Se Fins are partially oxidized to Bi_2_SeO_5_, forming an epitaxial high-*κ* gate dielectric of Bi_2_SeO_5_, and finally constructing a FinFET structure as shown in Fig. 6h [110].

#### GAA FETs and MBC FETs

The core advantage of the GAA structure is that the gate surrounds the channel from all sides, greatly improving gate control capability and effectively suppressing short-channel effects. The GAA structure reduces power by nearly half (45% lower) compared to 5 nm FinFET (where the gate only wraps the channel on three sides), improves performance by 23%, and reduces area by 16% [117]. For 2D semiconductors, the channels can be fabricated into ultra-thin nanosheets (thickness <2 nm), and the gate dielectric surrounds the nanosheets to form a ‘nanotube’ structure, further enhancing gate control capability.

Tang *et al.* proposed Bi_2_O_2_Se GAAFETs, as shown in Fig. 6i. Based on the epitaxial integration of 2D semiconductor Bi_2_O_2_Se and native high-*κ* dielectric Bi_2_SeO_5_ (*κ* ≈ 21), a core-shell structure is constructed through a low-temperature monolithic three-dimensional (M3D) process. The Bi_2_SeO_5_ dielectric completely encapsulates the Bi_2_O_2_Se channel, forming an atomically flat vdW interface. With the GAA architecture, extreme electrostatic control is achieved. The device with a channel length of 30 nm operates at a voltage as low as 0.5 V, with *I*_on_ exceeding 1 mA/μm, an intrinsic delay of 1.9 ps, and an energy-delay product of 1.84 × 10^−27^ Js/μm, meeting the requirements of IRDS 2028 advanced nodes [109].

MBCFET is a novel structure proposed by Samsung Electronics for the 3-nanometer node. Its core is to replace the source-drain PN junctions of traditional MOSFETs with ‘bridge channels’, significantly reducing parasitic capacitance and resistance. Samsung directly grows 2D channels of MoS_2_ (NMOS) or WSe_2_ (PMOS) on the multi-bridge dielectric structure, as shown in Fig. 6j. Utilizing the multi-bridge architecture to achieve channel width modulation, wafer-level integration can be completed without transfer processes, balancing process stability and device performance. The MoS_2_ MBCFET achieves an on/off ratio of 6 × 10^3^, realizes current control through channel width modulation (0.3–2.8 μm), has a leakage current lower than 100 pA, and a device yield of 80% on 200 mm wafers [111].

IMEC used MoS_2_/WS_2_ monolayer 2D materials as channels to achieve encapsulation of high-*κ* dielectric (HfO_2_), as shown in Fig. 6k. A stacked nanosheet structure was constructed through a double-gate or GAA architecture. The double-layer stacked device with a channel length of 40 nm achieves an on-state current of 451 μA/μm, an on/off ratio exceeding 10^9^, a transconductance (*g*_m_) of 350 μS/μm for the double-gate device, and a contact resistance lower than 440 Ω μm. A device yield of 96.59% is achieved, supporting process migration from double-gate devices to stacked nanosheets, laying the foundation for subsequent 2D CFETs [112].

#### Complementary field-effect transistors

CFETs achieve complementary logic functions by vertically stacking n-type and p-type FETs, which significantly reduces the cell area, making it a core scheme for high-density integration of angstrom nodes. Intel vertically stacks NMOS and PMOS to replace the traditional planar side-by-side structure, achieving a 50% reduction in standard cell area, as shown in Fig. 6l. The subthreshold swing of the device with a gate length of 75 nm is as low as 65–69 mV/dec, and DIBL<50 mV/V; at a 48 nm pitch, the on-state current (*I*_on_) is 30% higher than that of traditional planar CMOS [113]. IMEC focused on the key middle-of-line (MOL) process of CFETs, developed high-aspect-ratio tungsten filling and etch-back technologies to solve the source-drain contact problem of vertically stacked devices, and realized a stacked contact structure of BC-isolation layer-TC, supporting large-scale integration of monolithic CFETs, as shown in Fig. 6m [114]. TSMC fabricated monolithic CFETs with a 48 nm Pitch based on a nanosheet architecture. NMOS (top layer) and PMOS (bottom layer) are vertically stacked, and three innovative structures—middle dielectric isolation, internal spacer, and source-drain isolation—are adopted to solve the leakage and interconnection problems of upper and lower layer devices, meeting the density and performance requirements of industrial-grade advanced nodes (approaching 1 nm). For devices with a gate length of 15 nm and a gate pitch of 48 nm, the SS of NMOS/PMOS is 75/73 mV/dec, and DIBL is 50/45 mV/V, respectively, with gate control capability superior to traditional FinFETs [118].

The interlayer vdW interactions of 2D semiconductors make them ideal materials for CFET stacking: n-type (such as MoS_2_, WS_2_, and Bi_2_O_2_Se) and p-type (such as WSe_2_ and SnS_2_). 2D semiconductors can be vertically stacked without damage through vdW transfer, with low interface parasitic resistance. Liu *et al.* prepared n-MoS_2_ and p-WSe_2_ FETs on different substrates, then stacked the p-type FETs on top of the n-type FETs through vdW transfer, and used ALD-grown HfO_2_ as the interlayer dielectric to form a CFET structure, as shown in Fig. 6n [115]. Pendurthi *et al.* fabricated the 2D CFET vertically stacked n-WSe_2_ (Ni/Pt contacts) and p-WSe_2_ (Pd/Au contacts) FETs, and used Ni/Au-filled vias to realize interconnection between the bottom n-type FET and the top p-type FET, as shown in Fig. 6o [116].

In the future, for angstrom nodes, the research and development of advanced structure 2D transistors need to focus on three directions: first, develop 2D semiconductor preparation technologies compatible with existing silicon-based processes, increasing the wafer-level yield to above 80%; second, further reduce the Pitch to below 30 nm through multi-stack GAA, 3D CFET and other structures to achieve improved integration density; third, realize the heterogeneous integration of 2D transistors and silicon-based circuits to construct ‘2D-silicon’ hybrid chips, meeting the requirements of high-performance computing.

## 2D MATERIALS-BASED CIRCUITS AND INTEGRATED SYSTEMS

In recent years, 2D materials have achieved remarkable advancements in fabrication processes, transfer techniques, and device performance optimization. However, the superior performance of individual devices is difficult to directly translate into system-level circuit advantages, and their feasibility in large-scale integrated circuits is crucial for evaluating whether 2D materials can complement or replace silicon-based technologies to sustain Moore’s law. This section systematically reviews the latest progress and key challenges of 2D materials in circuit integration, focusing on three core directions: 2D materials-based basic circuit units, 2D large-scale logic and memory chips, and 3D integration technologies.

### 2D materials-based basic circuit units

**Figure 7.**
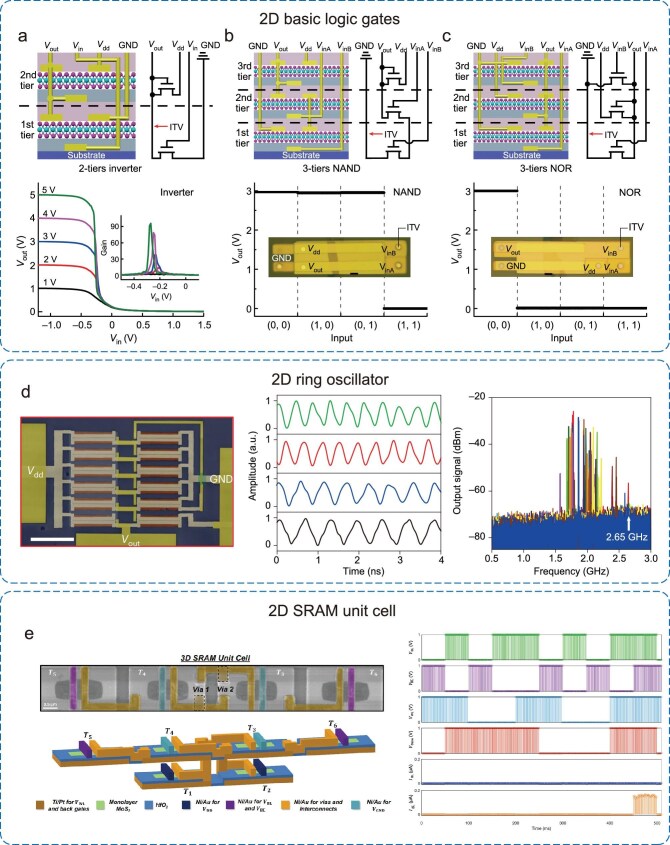
2D materials-based basic circuit units. (a) Schematic, circuit, and characteristics of 2-tiers inverter based on n-type MoS_2_ transistors [120]. (b and c) Schematics, circuits, and characteristics of 3-tiers MoS_2_ NAND and 3-tiers MoS_2_ NOR [120]. Copyright 2024, Springer Nature. (d) Layout and performance of MoS_2_ five-stage ring oscillator [121]. Scale bar: 20*μ*m. Copyright 2023, Springer Nature. (e) Layout and characteristics of n-type MoS_2_ SRAM [124]. Copyright 2025, Springer Nature.

In 2011, Radisavljevic *et al.* fabricated the first 2D ‘pseudo-inverter’ using exfoliated monolayer MoS_2_ transistors, by connecting the gate electrode of the load transistor to the drain electrode of the switch transistor. The ‘pseudo-inverter’ demonstrated a voltage gain over 4 with a supply voltage of 2.0 V and an input voltage range of −4.0 V to +4.0 V [119]. In 2024, Das *et al.* constructed a CMOS inverter using n-type and p-type WSe_2_ transistors [116], with a supply voltage *V*_DD_ of 3.0 V and an input voltage range of 0–3.0 V. This inverter achieved a voltage gain exceeding 80, attributed to the excellent electrostatic control of WSe_2_ devices that suppresses off-state leakage. Also in 2024, Liu *et al.* developed a bilayer pseudo-inverter based on MoS_2_ via a low-temperature vdW lamination of prefabricated circuit layers (Fig. 7a) [120]. This MoS_2_-based inverter worked at a supply voltage of 1.0 V and an input voltage range of −1.0–1.5 V; the low operation voltage could effectively reduce the circuits power consumption. Since transistors based on 2D materials do not strictly require process steps such as ion implantation doping and high-temperature activation, they not only inflict less damage on the devices but also offer a superior thermal budget which is crucial for large-scale circuit manufacturing.

Using the aforementioned method, Liu *et al.* further realized stable-performance NAND and NOR circuits working with a supply voltage of 3.0 V and an input voltage range of 0–3.0 V [120], as shown in Fig. 7b and c, laying the foundation for the development of high-density 3D computing systems. In 2024, Das *et al.* reported multi-layer NAND and NOR circuits based on n-type and p-type WSe_2_ [116], the circuits working with a supply voltage *V*_DD_ of 3.0 V and an input voltage range of 0–3.0 V, promoting M3D integration technology to break through the limitations of traditional planar scaling. Additionally, in 2023, Wang *et al.* demonstrated a five-stage ring oscillator operating at 2.65 GHz under a supply voltage *V*_DD_ of 3.1 V (Fig. 7d) [121], highlighting the great application value of 2D materials at the level of basic unit circuits.

Static random-access memory (SRAM), serving as high-speed cache in ultra-large-scale integrated circuits such as CPUs and GPUs, has performance that highly depends on the intrinsic properties of semiconductor materials. Compared with traditional silicon-based materials (bandgap ∼1.12 eV), 2D materials such as MoS_2_ (∼1.8 eV), WS_2_ (∼2.0 eV), and WSe_2_ (∼1.6 eV) exhibit wider bandgap ranges, which significantly suppress thermally excited carrier concentrations at room temperature, thereby providing a vital material basis for constructing novel SRAM cells with ultra-low leakage currents and static power consumption. The first 2D SRAM was fabricated using exfoliated bilayer MoS_2_, with both depletion-mode (negative threshold voltage) and enhancement-mode (positive threshold voltage) devices displaying on/off ratios exceeding 10^7^ and on-state currents over 23 μA/μm at *V*_gs_ = 2.0 V and *V*_ds_ = 1.0 V [122].

In 2018, the first WSe_2_-based CMOS SRAM was constructed by combining chemically doped p-type transistors and electrostatically doped n-type transistors, enabling stable operation at a low voltage of 0.8 V and further reducing circuit power consumption [123]. In 2021, Wang *et al.* proposed 4-transistor-2-resistor and 3-transistor-3-resistor SRAMs, which realized on-chip XNOR/XOR and NAND/NOR computing functions.

Vertical stacking of SRAM is the key to further improving integration density. In 2025, Das *et al.* fabricated field-effect transistors using monolayer MoS_2_ and achieved 2-layer and 3-layer stacked 6T SRAM cells via monolithic 3D integration with a supply voltage of 1.0 V (Fig. 7e) [124]. This team successfully produced a 1-kilobit 2-layer 3D SRAM array comprising 6144 MoS_2_ FETs, occupying only 0.0251 mm^2^, demonstrating exceptionally high integration density.

### Large-scale 2D logic, memory, and bioinspired chips

**Figure 8.**
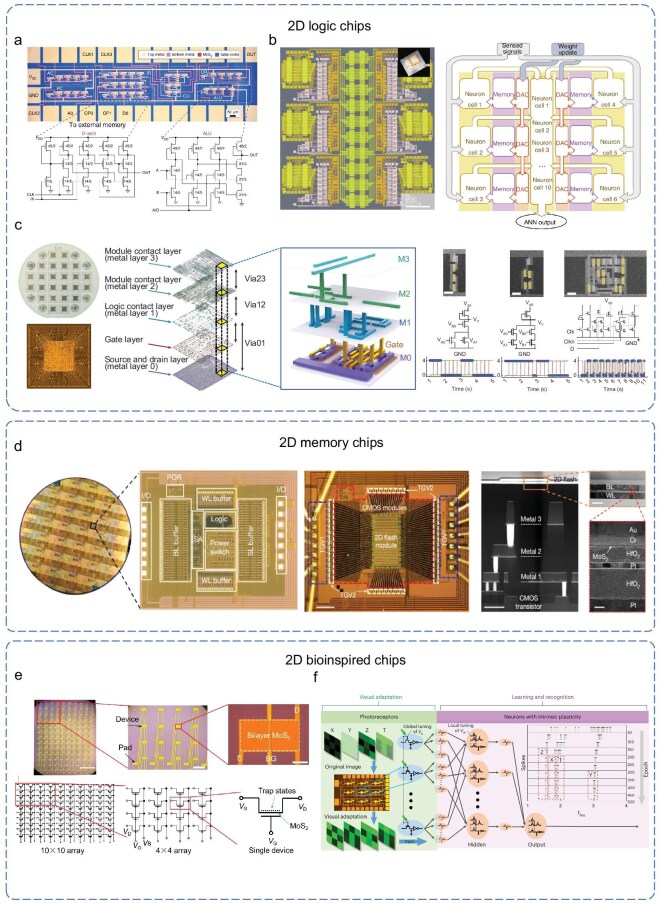
Large-scale 2D logic, memory and bioinspired chips. (a) Layout and circuit diagrams of the first 1-bit MoS_2_ microprocessor [125]. ALU: arithmetic logic unit; CU: control unit; IR: instruction register; PC: program counter. Copyright 2017, Springer Nature. (b) Architecture and circuit of artificial neural network chips [126]. DAC: digital-to-analog converter; ANN: artificial neural network. Copyright 2021, Science China Press Published by Elsevier B.V. (c) Multilayer structure, circuit schematics, and system diagram of 2D logic chips RV32-WUJI [128]. Scale bars: 10μm. Copyright 2025, Springer Nature. (d) Layout and structure of 2D flash chips [131]. Scale bars: 250μm (left); 1μm (middle); 200 nm (top right); 5 nm (bottom right). SA: sense amplifier; POR: power-on reset. Copyright 2025, Springer Nature. (e) Schematic of a bioinspired vision sensor based on the MoS_2_ phototransistor array [132]. Scale bars: 4 mm (left); 1 mm (middle); 100 μm (right). Copyright 2022, Springer Nature. (f) Device structure and functional demonstration of the MoS_2_-based biologically inspired artificial neuron module [133]. Copyright 2025, Springer Nature.

In 2017, Wachter *et al.* reported the first 1-bit microprocessor based on 2D MoS_2_ (Fig. 8a), integrating 115 transistors, working at an operation voltage of 5.0 V capable of executing user-defined programs, performing logical operations, and communicating with peripherals [125]. In 2022, Bao *et al.* realized an artificial neural network based on 2D transistors (Fig. 8b), integrating 818 transistors with multiply-and-accumulate, storage, and activation functions, paving the way for 2D semiconductors in artificial intelligence computing [126]. In 2025, Das *et al.* developed a 1-bit computer based on 2D material inverters with a supply voltage of 3 V [127]. The computer is capable of NAND, NOR, XOR, MUX, and DFF functions, with an operating frequency of 25 kHz and power consumption as low as 100 pJ. In the same year, Zhou *et al.* successfully developed a RISC-V 32-bit microprocessor RV32-WUJI based on 2D semiconductors (Fig. 8c) [128], integrated on a 4-inch sapphire wafer with 5931 MoS_2_ transistors in a 6 mm × 6 mm chip area, operating at 4 V supply voltage and 1 kHz frequency with a power consumption of only 0.43 mW, marking a significant milestone in large-scale integration of 2D semiconductors.

2D materials have also made breakthrough progress in memory circuits, with studies indicating that flash devices based on 2D materials can achieve read/write speeds as low as 400 ps [129]. Recent advancements in fabricating large-scale 2D memory circuits include: In 2020, Marega *et al.* reported floating-gate field-effect transistors based on MOCVD-synthesized large-scale MoS_2_, enabling basic processor computing modules such as half-adders and three-input NAND gates without complex current–voltage conversion circuits, offering low-power advantages [130]. In 2025, Liu *et al.* integrated 2D flash memory cells with mature CMOS platforms, realizing a fully functional 2D NOR flash chip with a yield of 94.34% (Fig. 8d) [131]. This chip supports 8-bit instructions, 32-bit parallel operations, and random access, with a clock frequency of 5 MHz and data retention of 10 years at 54.8°C, accelerating the transition of 2D materials from laboratory to industrialization.

2D materials are also widely used to construct bioinspired circuits, with numerous breakthroughs achieved in this field in recent years. In 2022, Chai *et al.* developed a bioinspired vision sensor based on an array of MoS_2_ phototransistors as shown in Fig. 8e [132]. Its core is to emulate the scotopic and photopic adaptation functions of the human retina, and the effective perception range of this device array reaches up to 199 dB, far exceeding that of traditional silicon-based CMOS arrays (70 dB) and the human retina (160 dB). In 2025, Chai *et al.* reported a biologically inspired artificial neuron module based on MoS_2_ that successfully simulates neuronal intrinsic plasticity and visual adaptation functions [133]. The biologically inspired neural network constructed based on this module can achieve visual adaptation and feature recognition as shown in Fig. 8f, providing an efficient solution for edge intelligence hardware. This study for the first time achieved the integration of intrinsic plasticity and visual adaptation functions based on MoS_2_ material, greatly simplifying the hardware architecture of biologically inspired visual systems.

### 3D homo-integration of 2D circuits

In conventional planar circuit designs, the horizontal interconnect distances between functional layers range from micrometers to millimeters, resulting in signal delays and elevated power consumption. 3D integration can shorten the interlayer interconnect lengths to the nanometer scale, effectively mitigating power and delay issues while significantly enhancing integration density and reducing structural complexity.

Traditional silicon-based 3D integration faces severe thermal budget and power consumption challenges: the high activation temperatures inevitably degrade circuit performance; the stacked structures impede heat dissipation, and the high power density of integrated circuits causes rapid heat accumulation [134]. In contrast, 2D material-based circuits do not require doping processes, enabling the processing temperature to be reduced below 400°C; 2D materials also possess wider bandgaps, enabling circuits with lower off-state currents and static power consumption, which can effectively alleviate the heat dissipation pressure of 3D stacked circuits [42,135,136].

**Figure 9.**
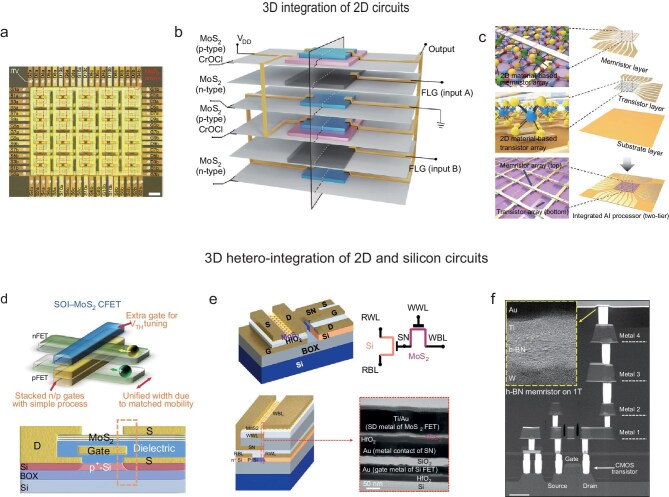
3D integration and hetero-integration of 2D circuits. (a) Optical image of 10-tier MoS_2_ M3D system [120]. Copyright 2024, Springer Nature. (b) Schematic of 3D-integrated logic gates [137]. Copyright 2024, Springer Nature. (c) M3D integration of 2D material-based memristors and transistors [138]. Copyright 2023, Springer Nature. (d) Schematic of SOI–MoS_2_ heterogeneous 3D-stacked CFET [139]. Copyright 2022, Springer Nature. (e) Schematics and circuits of Si-MoS_2_ heterogeneous embedded DRAM [140]. RWL: read word line; WWL: write word line; SN: storage node; RBL: read bit line; WBL: write bit line. Copyright 2024, Springer Nature. (f) STEM images of hybrid 2D–CMOS memristive microchips [141]. Scale bar: 600 nm. Copyright 2023, Springer Nature.

In 2024, Liu *et al.* proposed a low-temperature monolithic 3D integration method based on vdW lamination [120], achieving vertical stacking of 10-layer 2D MoS_2_ semiconductor circuits (total thickness ∼8 μm), as shown in Fig. 9a. Compared with traditional planar structures, the stacked 3D structure achieves a 10× higher logic density and eliminates horizontal interconnect delays, translating device-level advantages into system-level density and energy efficiency. Also in 2024, Han *et al.* achieved controllable modulation of carrier polarity in 2D semiconductors through vdW interface coupling [137], fabricating 6-layer stacked inverters, 14-layer stacked NAND gates, and SRAMs (Fig. 9b), verifying the feasibility of vertical integration. In 2023, Kang *et al.* implemented a neuromorphic system based on 2D materials using M3D technology [138]. The system consists of 3 layers of 2D transistor arrays and 3 layers of 2D memristor arrays, adopting WSe_2_/h-BN memristors and MoS_2_ transistors to achieve wafer-free stacking (Fig. 9c). This system supports near-sensor/in-sensor computing architectures, capable of AI tasks such as DNA motif discovery. The ultra-thin structure of 2D materials facilitates the stacking of multi-functional layers (sensing, storage, and computing), laying the foundation for novel computing paradigms such as in-memory computing. Specifically, the co-integration of memristors (storage) and transistors (logic) in vertical layers reduces data movement between components, improving energy efficiency, directly translating 2D materials’ vertical stacking advantage into a novel, high-efficiency computing architecture.

### 3D hetero-integration of 2D and silicon circuits

2D materials, with atomic-scale thickness, absence of dangling bonds, and superior gate control, maintain high on/off ratios (>10^6^) even at 1 nm gate lengths, offering possibilities for continued device scaling. The hetero-integration of 2D materials and silicon can effectively improve space utilization, shorten interconnection length, and reduce delay and energy consumption.

In 2022, Ling *et al.* reported a heterogeneous CMOS inverter based on MoS_2_ nFETs and SOI pFETs (Fig. 9d) [139]. This device achieved a voltage gain of 142.3 at a 3 V supply voltage and power consumption of only 64 pW at 0.1 V, providing an innovative solution to the longstanding mobility mismatch between n-type and p-type transistors in traditional silicon-based CMOS inverters.

In 2024, Zhou *et al.* constructed a 2T-0C DRAM structure via heterogeneous integration of MoS_2_ FETs with silicon-based FETs (Fig. 9e) [140]. The fabricated devices exhibited data retention times up to 6000 seconds, with a write pulse width as low as 5 ns. This work, by vertically stacking MoS_2_ functional layers on top of silicon transistors, doubled integration density and overcame parasitic capacitance and stress limitations in traditional silicon-based 3D integration. MoS_2_’s ultra-low off-state current minimizes charge leakage from the DRAM storage node, significantly extending data retention time and reducing refresh frequency.

In 2023, Lanza *et al.* transferred multi-layer h-BN to the back-end interconnect layers of CMOS chips, successfully constructing a 5 × 5 crossbar array of single-transistor-single-memristor (1T1M) cells on a 2 cm × 2 cm silicon-based CMOS chip, as shown in Fig. 9f [141].

This 2D material-CMOS hybrid memristive microchip can implement in-memory logic operations such as ‘OR’ and ‘implication’, and its memristor cells exhibit spike-timing-dependent plasticity characteristics. This study represents the first high-density coupling of small-sized (0.053 μm^2^) 2D material memristors with CMOS, offering an effective route to address core challenges of low integration density in 2D material devices.

Despite the significant progress in circuit integration with 2D materials, two key issues remain unresolved: maintaining performance advantages in large-scale integration scenarios and designing reliable interconnects for stable, cost-effective practical chips. In the future, ongoing innovations in material preparation, integration processes, and design methodologies position 2D materials as a central direction in post-Moore electronics, injecting new vitality into the sustained evolution of integrated circuits.

## OUTLOOK

For integrated electronics targeting sub-1-nm nodes, the development of 2D materials is transitioning from experimental exploration toward systematic engineering implementation, with core challenges spanning the materials, device, and system levels. While remarkable scientific progress has been achieved, significant bottlenecks remain before large-scale manufacturing can be realized, particularly concerning wafer-to-wafer variability, defect density control, yield, thermal budget, and compatibility with existing CMOS process flows.

At the materials level, the uniformity and defect density of wafer-scale 2D materials remain critical factors limiting performance enhancement. Although current CVD and MBE techniques can achieve large-area coverage, microscopic defects such as thickness variations, grain boundaries, and vacancies still significantly degrade carrier mobility and device reliability. These variations introduce substantial wafer-to-wafer and within-wafer variability, which directly impacts circuit-level yield and reproducibility. Future efforts should focus on atomic-scale uniform growth and controllable defect engineering through self-limiting epitaxy, interface nucleation control, and *in situ* characterization of defect–transport relationships, as well as the development of statistical metrology and process control methodologies analogous to those used in advanced silicon manufacturing.

At the device level, contact resistance and interface-state engineering remain major bottlenecks. Fermi-level pinning at metal–2D semiconductor interfaces, dielectric polarization impurities, and interfacial traps severely degrade device uniformity and subthreshold characteristics. The use of phase-change metal contacts, interface interlayers, and high-quality passivation dielectrics can effectively improve contact performance and carrier mobility; however, reliable models for band alignment design and high-field degradation mechanisms are still lacking. In addition, the absence of low-*κ* spacer processes compatible with 2D materials directly limits transistor switching speed. For 2D semiconductor devices to move from lab to fab, variability, reliability, and long-term stability must be addressed. Key efforts include wafer-scale growth with uniform, controllable atomic layers and defect suppression, as well as developing BEOL-compatible low-temperature processes (<400°C) for contacts and dielectrics. Key efforts also include advancing passivation and encapsulation to resist operational degradation. Synergizing material synthesis, process integration, and mechanism optimization will unlock 2D devices’ potential for beyond-silicon electronics.

At the system level, the compatibility of 2D materials with CMOS processes and their scalability toward large-volume manufacturing are prerequisites for industrial adoption. Key issues include contamination control, alignment accuracy in lithography, integration with existing metallization stacks, and reliability under standard manufacturing environments. Moreover, wafer-to-wafer variability in material properties remains a major concern for high-volume production. Future efforts should focus on the development of key processes such as low-temperature epitaxy, wafer-scale dry bonding, and automated transfer technologies, as well as the establishment of multiscale frameworks for reliability and statistical uniformity evaluation encompassing DTCO, yield, and long-term stability considerations. Hybrid integration strategies—such as selective-area growth on patterned wafers or vdW wafer bonding—may offer more realistic near-term pathways toward industrial implementation.

Looking ahead, the atomic-scale thickness and tunable band structure of 2D materials provide a novel physical platform for angstrom logic devices, with significant potential in AI chips and three-dimensional heterogeneous integration. To push 2D integrated electronics further toward angstrom-node device applications, exploring emerging material systems beyond traditional TMDs is imperative; in this context, the vdW-layered MoSi_2_N_4_ family, endowed with tailorable electronic properties and scalable synthesis capabilities, emerges as a particularly promising candidate for next-generation transistors and non-volatile memory devices [142]. By integrating logic, memory, and optoelectronic functionalities via vdW heterostructures, 2D materials are poised to drive the evolution of information processing architectures from conventional silicon-based topologies toward materials-driven intelligent systems, enabling ultra-low-power and high-density computing in the post-Moore era. Advancing 2D materials to sub-1-nm technology nodes presents a full-chain challenge spanning material synthesis, device physics, and system integration. Its development trajectory is not a simple silicon replacement but serves as a key enabling technology, opening new application avenues through heterogeneous integration.
